# The use of traditional medicine practitioner services for childhood illnesses among childbearing women: a multilevel analysis of demographic and health surveys in 32 sub-Saharan African countries

**DOI:** 10.1186/s12906-023-03972-3

**Published:** 2023-04-29

**Authors:** Peter Bai James, Razak M. Gyasi, Ossy Muganga Julius Kasilo, Jon Wardle, Abdulai Jawo Bah, George A. Yendewa, Amos Deogratius Mwaka

**Affiliations:** 1 National Centre for Naturopathic Medicine, Faculty of Health, Southern Cross University, Lismore, NSW 2480 Australia; 2 Faculty of Pharmaceutical Sciences, College of Medicine and Allied Health Sciences, University of Sierra Leone, Freetown, Sierra Leone; 3 African Population and Health Research Center (APHRC), Nairobi, Kenya; 4 WHO Regional Office for Africa, Universal Health Coverage Life Course Cluster, Brazzaville, Republic of Congo; 5 Institute for Global Health and Development, Queen Margaret University Edinburg, Musselburgh, Scotland, UK; 6 Division of Infectious Diseases and HIV Medicine, University Hospitals Cleveland Medical Center, Cleveland, OH 44106 USA; 7 Johns Hopkins Bloomberg School of Public Health, Baltimore, MD 21205 USA; 8 Department of Medicine, Faculty of Medicine, Gulu University, Gulu, Uganda

**Keywords:** Traditional medicine, Sub-Saharan Africa, Diarrhea, Fever, Cough, Child health

## Abstract

**Background:**

Insights into the use of traditional medicine practitioners (TMP)-for common childhood diseases such as diarrhea and respiratory infections are important to understand the role of Traditional Medicine (TM) in reducing the increasing childhood morbidity and mortality in sub-Saharan Africa (SSA). However, a comprehensive picture of TMP utilisation and its associated factors for childhood illness in SSA is lacking. This study aimed to estimate the prevalence of the use of traditional medicine practitioner services to treat childhood illnesses among women with children under five years old and to identify individual and community-level factors associated with TMP use in SSA.

**Methods:**

The analysis used Demographic and Health Surveys (DHS) dataset collected between 2010 and 2021 among 353,463 under-fives children from 32 SSA countries. Our outcome variable was the use of TMP for childhood illness, defined as having diarrhoea or fever/cough or both. Using STATA v14, we employed the random effect meta-analysis to estimate the pooled prevalence of TMP use for childhood illness and a two-level multivariable multilevel modelling to determine the individual and community-level factors associated with consultation of a TMP.

**Results:**

Approximately [2.80% (95%CI: 1.88–3.90)] women who sought healthcare for childhood illnesses utilised the service of a TMP with the highest occurring in Cote d’Ivoire [16.3% (95%CI: 13.87–19.06)] and Guinea (13.80% (95%CI: 10.74–17.57)] but the lowest in Sierra Leone [0.10%(95%CI:0.01–1.61)]. Specifically, approximately [1.95% (95%CI: 1.33–2.68)] and [1.09% (95%CI:0.67–1.60)] of women sought the service of a TMP for childhood diarrhea and fever/cough, respectively. Women with no formal education [AOR = 1.62;95%CI:1.23–2.12], no media access [AOR = 1.19;95%CI:1.02–1.39), who lived in a male-headed household [AOR = 1.64;95%CI:1.27–2.11], without health insurance [AOR = 2.37;95%CI: 1.53–3.66], who considered it a problem getting permission to visit a health facility [AOR = 1.23;95%CI:1.03–1.47] and who perceived the size of their children at birth to be above average[AOR = 1.20;95%CI:1.03–1.41] had higher odds of using TMP for childhood illnesses.

**Conclusions:**

Although the prevalence of TMP for childhood illnesses appeared low, our findings highlight that TMPs continue to play a critical role in managing childhood illnesses in SSA. It is essential that policymakers and service providers should incorporate the potential role of TMPs in the design, review and implementation of child health policies in SSA. Also, the interventions for curtailing childhood illnesses should be focused on the characteristics of women who use TMPs for childhood diseases identified in our study.

**Supplementary Information:**

The online version contains supplementary material available at 10.1186/s12906-023-03972-3.

## Background

Child health and survival continue to be a global health problem despite the progress made globally in the past two decades [1]. Even though there was a 59%reduction in under-five mortality from 93 deaths per 1,000 live births in 1990 to 38 in 2021, five million children globally still lost their lives before their fifth birthday in 2021 [1]. Sub-Saharan Africa (SSA) bears the highest burden of under-five mortality, with one child in 13 dying before his or her fifth birthday, mainly from preventable causes [1]. However, such an estimate does not fully provide a nuanced picture of countries on the continent [1]. While countries such as Rwanda, Liberia, Madagascar, and Malawi have recorded a greater than 60% decrease in under-five mortality between 1990 and 2018, the majority are still lagging [2]. Indeed, infectious diseases such as pneumonia (15%), diarrhoea (8%)and malaria (5%) are the leading cause of under-five mortality in SSA [3]. Diarrhea is the leading cause of one out of every six deaths of children under five globally, and mostly results from eating or drinking contaminated food or drink [3]. Pneumonia accounts for one in seven deaths, with 50% of those deaths occurring in SSA. Pneumonia in children is caused by a bacteria, viruses and fungi. The most common infectious agents are Streptococcus pneumoniae, Haemophilus influenzae type b and Respiratory syncytial virus [3]. Reducing the high burden of childhood illness is an important aim of Sustainable Development Goal (SDG) number three, which sets to reduce under-five mortality to as low as 25 deaths per 1000 live births by 2030 [4]. Although such a SDG goal is achievable, there are concerns that such a target will not be achieved due to weak health systems in SSA and their poor positioning to adequately respond to the high burden of childhood illness [5]. Access to healthcare for childhood illness has been identified as a significant challenge in SSA due to long distances to health facilities, low income, dissatisfaction with previous healthcare, and health illiteracy [6, 7]. Such factors have pushed many people to seek other healthcare options such as traditional medicine (TM) [8].

Traditional medicine is a set of knowledge, skills, and practices based on the theories, beliefs, and experiences indigenous to different cultures, whether explicable or not, used in the maintenance of health as well as in the prevention, diagnosis, improvement, or treatment of physical and mental illness [9]. Traditional medicine has been practised in SSA since time immemorial, and its use is widespread across countries on the continent [8]. Traditional medicine popularity is attributed to its philosophical alignment with African culture and tradition, accessibility and modern healthcare not meeting individual health needs [8]. The role of TM in global health became more apparent than before, with an upsurge in its use over the past decades. Such an uptake has attracted decision-makers, researchers, and health providers in a quest to understand how TM can be leveraged to promote public health. Despite the progress that has been made in developing policies, enhancing research, cultivation of raw materials, regulating TM products and practitioners and promoting collaboration between traditional medicine practitioners (TMP) and conventional medicine practitioners, TM integration into the main healthcare system still faces many challenges in SSA countries [10]. Some of these challenges include a lack of translation of political will and a lack of scientific evidence in the safety and efficacy of many TM products and practices due to funding for the production, research, and development of TM [8].

Notwithstanding the challenges associated with TM in general, TMPs are known to be respected in African society, and their role in providing primary healthcare is well documented [11–14]. Also, many community studies in Africa have reported TMP utilisation in managing childhood illness and the factors associated with such use. For instance, community studies in Mali and Ethiopia reported that approximately half of the caretakers sought the service of TMP for their children’s illnesses [15, 16]. Also, 6.4% of caregivers sought the service of a TMP for childhood diarrhea in the rural areas of Burkina Faso [17], while 18.5% first consulted with a TMP for childhood malaria in Western Ethiopia [18]. Furthermore, a qualitative study in Gabon and Benin reported that traditional healers were consulted for childhood illness, especially folk illness [19]. A review of the available literature on health-seeking behaviour for childhood illness in Africa indicates that studies that specifically looked at the role of TMP in managing childhood diseases in Africa are community-based quantitative or qualitative studies or reviews [15–21]. Although quantitative multi-country studies have been conducted on the healthcare-seeking behaviour for childhood illness in Africa, all of them have so far focussed on the utilisation of conventional healthcare, i.e., utilisation of public and private healthcare facilities [7, 22, 23]. To our knowledge, we have not seen any multi-country study that has explored TMP utilisation for childhood illness in Africa. Thus, our study fills that knowledge gap in the contemporary literature. Also, our study will provide insights into TMPs’ contribution to managing childhood illnesses in Africa and the individual and community level factors that may serve as facilitators and barriers to seeking the service of a TMP. Thus, our study has two aims, and they are 1) to estimate the prevalence of the use of TMP services to treat childhood illnesses (diarrhea or fever/cough or both) among women with children under the age of five and 2) to determine the individual and community-level factors associated with seeking the service of a TMP using Demographic and Health Surveys (DHS) data from 32 African countries.

## Methods

### Study design and data source

We used secondary cross-sectional data from the DHS from 32 countries in SSA conducted between 2010 and 2021. The included countries are Angola, Benin, Burkina Faso, Burundi, Chad, Cameroun, Comoros, Congo Democratic Republic, Ethiopia, Gambia, Ghana, Guinea, Gabon, Ivory Coast, Kenya, Liberia, Mali, Madagascar, Malawi, Mauritania, Mozambique, Namibia, Nigeria, Niger, Rwanda, Senegal, Sierra Leone, South Africa, Togo, Uganda, Zambia and Zimbabwe. We chose the countries if the DHSs were conducted between 2010 and 2021, and their datasets included the outcome and explanatory variables of interest. The DHS is a nationally representative survey conducted in 85 low- and middle-income countries to collect population, health, and nutrition data using a standardised questionnaire. The countries can add modules significant to their respective local health context [24]. Briefly, the DHS uses a two-stage stratified sampling strategy. The first stage involves determining Enumeration Areas (EA) from the census files. The second stage involved the selection of a sample of households taken from an updated list of households in each of EA identified in the first stage [25]. Details of the sampling procedure are fully described elsewhere [26]. The DHS questionnaire is usually designed in the’country’s official language but is often translated into the major local languages [24, 26]. The recoded children files (KR) for each country were used for our analysis, and they include all women who were caregivers of children under five or gave birth within the five years prior to conducting the respective surveys. We used data from a sample of 353,463 mothers of under-fives children during the survey. The detail of the countries involved, the year the DHS was conducted, and their respective samples are shown in Table 1. Of the 353,463 women interviewed, 49,529 and 105,187 children had diarrhea and fever/cough, respectively. Among women whose children had diarrhea and fever/cough, 49,476 and 91,754 sought treatments (formal and informal) for childhood diarrhea and fever/cough, respectively, while 27,060 women sought one form of treatment either for diarrhea or fever/cough or both for their children.

**Table 1:** Distribution of the study sample by country

Country	Year	Sample Used	% of sample used
Benin	2017–18	13,589	3.9
Burkina Faso	2010	15,044	4.4
Chad	2014–15	18,623	5.3
Gambia	2019–20	8362	2.2
Ghana	2014	5884	1.6
Guinea	2018	7951	2.3
Ivory Coast	2011–12	7776	2.1
Liberia	2019–20	5704	1.5
Mali	2018	9940	2.9
Niger	2012	12,558	3.8
Nigeria	2018	33,924	9.8
Angola	2015–16	14,322	3.8
Senegal	2019	6125	1.6
Sierra Leone	2019	9899	2.8
Togo	2013–14	6979	1.9
Burundi	2016–17	13,192	3.9
Cameroun	2018	9733	2.9
Comoros	2012	3149	0.9
Congo DR	2017–18	18,716	5.3
Ethiopia	2016	10,641	3.1
Gabon	2012	6067	1.5
Kenya	2014	20,964	5.6
Madagascar	2021	12,499	3.5
Malawi	2015–16	17,286	5.0
Mozambique	2011	5178	1.6
Mauritania	2019–21	11,628	3.3
Namibia	2013	5046	1.4
Rwanda	2019–20	8092	2.4
South Africa	2016	3548	1.0
Uganda	2016	15,522	4.4
Zambia	2018	9959	2.8
Zimbabwe	2010–11	5563	1.6
Total		353,463	100.0

### Study variables

#### Outcome variable

Our outcome variable was the use of a traditional medicine practitioner (TMP) for childhood illness. The outcome variable was based on the following questions: “Did you seek advice or treatment for 1) diarrhea from any source?” and 2) fever/cough from any source?” Those who answered “Yes” were asked: “Where did you seek advice or treatment for 1) diarrhea? And 2) fever/cough?” Three main options were provided, including “Public Sector”, “Private Sector”, and “Other Sources”. The sub-options under “Other Sources” were shop, traditional medicine practitioner, market, itinerant drug seller and others, which the respondents were asked to specify. Respondents were asked to indicate “Yes”, or “No”, or “I Don’t Know” to each of the sub-options. This analysis was interested in women’s consultation of a TMP for their child who had diarrhea or fever/cough. Those who answered “Yes” to the sub-option “traditional medicine practitioner” for diarrhea and fever/cough were considered to have sought the services of a TMP for those conditions. Thus, the main outcome variable (consultation of a TMP for childhood illness) was derived as a composite variable from the responses to the two sub-options for traditional medicine practitioner, i.e. “consultation of a TMP for diarrhea (yes vs no) and “consultation of a TMP for fever/cough (yes vs no). We excluded the “I don’t Know” category in computing our composite variable. In this study, respondents who answered “Yes” to any of the two sub-options for consultation of a TMP, i.e., “consultation of a TMP for diarrhea (yes vs no) and “consultation of a TMP for fever/cough (yes vs no) were considered to have sought the services of a traditional medicine practitioner for childhood illnesses and, were put in the category “Yes” and recoded 1. Those who answered “No” to the two sub-options for the consultation of a TMP were considered not to have sought the services of a traditional medicine practitioner for childhood illnesses and were put in the category “No” and recoded 0.

#### Independent variables

Individual- and community-level independent variables were derived from previous studies on seeking behaviour among children based on the DHS data [7, 15, 16, 19, 22, 23, 27–32]. Our analysis included four community-level variables (place of residence, household wealth, sex of household head and decision-making ability) and ten individual-level variables (maternal age, maternal education, marital status, working status, access to media, husband/partner educational status, birth order, twin status, parity, maternal health insurance status, problem accessing healthcare through difficulty with distance to health facility, difficulty in getting money for treatment, difficulty with getting permission to visit health facility and attitude toward wife beating). In addition, three childhood variables (age, sex, and size of the child at birth) were included. Table 2 provides a summary of the measurement of each variable.

**Table 2:** Distribution of the individual and community factors and TMP use for childhood illnesses among childbearing women in 32 African countries

**Characteristics**	**Variable**	**TMP use for childhood illnesses**			**TMP use for Diarrhea**			**TMP use for Fever /Cough**		
		**Yes n (%**^**a**^**)**	**No n (%**^**a**^**)**	**χ2 (*p*-value)**	**Yes n(%**^**a**^**)**	**No n(%**^**a**^**)**	**χ2 (*p*-value)**	**Yes n(%**^**a**^**)**	**No n(%**^**a**^**)**	**χ2 (*p*-value)**
Maternal Age	15-24 years	349(33.2)	8889(33.9)	4.03 (0.244)	468(35.9)	16,348(33.7)	4.56 (0.207)	411(29.5)	27,114(29.8)	1.32 (0.658)
	25-34 years	461(44.2)	11,964(46.1)		557(43.1)	22,136(46.2)		653(46.7)	42,847(47.7)	
	35-49 years	211(22.6)	5186(20.1)		257(20.9)	9710(20.1)		324(23.8)	20,405(22.5)	
Maternal Education	No formal education	576(59.0)	10,004(38.3)	51.23 (< 0.001)	692(56.5)	18,972(39.2)	**154.57 (< 0.001**)	820(61.4)	32,754(36.3)	**353.47 (< 0.001)**
	Primary education	302(28.3)	10,137(39.0)		389(29.4)	17,785(36.9)		373(25.3)	34,306(37.8)	
	Secondary plus	143(12.7)	5893(22.8)		201(14.1)	11,429(23.8)		195(13.3)	23,298(25.9)	
Marital Status	Single	49(4.6)	1594(5.8)	5.40 (0.136)	79(5.3)	3085(6.1)	4.57 (0.188)	62(4.4)	5482(5.8)	7.77 (0.068)
	Married /cohabitating	914(89.4)	22,527(86.8)		1127(88.9)	41,723(86.9)		1247(89.6)	78,308(87.0)	
	Divorced, separated/no longer living with partner	58(6.0)	1918(7.4)		76(5.7)	3386(7.0)		79(6.0)	6576(7.1)	
Residence	Urban	185(17.8)	7102(25.7)	30.40 (< 0.001)	266(20.1)	13,905(27.4)	**31.91 (< 0.001)**	256(18.2)	26,246(27.6)	**56.25 (< 0.001)**
	Rural	836(82.2)	18,937(74.3)		1016(79.9)	34,289(72.6)		1132(81.8)	64,120(72.4)	
Currently Working	Yes	686(71.2)	16,078(65.1)	14.65 (0.002)	812(66.3)	29,175(63.6)	3.61 (0.130)	958(71.4)	56,234(66.2)	**15.02 (0.003)**
	No	305(28.8)	8953(34.9)		435(33.7)	17,437(36.4)		403(28.6)	29,525(33.8)	
Household Wealth	Poorest	354(31.3)	7427(26.1)	42.83 (0 < .001)	456(33.8)	13,098(24.8)	**80.07 (< 0.001)**	480(30.5)	24,000(24.1)	**91.34 (< 0.001)**
	Poorer	259(26.7)	5994(23.7)		308(25.3)	10,901(23.2)		345(27.3)	20,220(22.8)	
	Middle	197(20.9)	5109(19.9)		242(18.5)	9554(20.1)		279(21.0)	17,946(20.3)	
	Richer	145(13.8)	4316(17.6)		187(14.7)	8254(18.1)		197(13.7)	15,513(18.0)	
	Richest	66(7.2)	3193(12.7)		89(7.8)	6387(13.8)		87(7.5)	12,687(14.7)	
Sex of Household Head	Male	870(85.6)	20,401(78.8)	25.875 (< 0.001)	1062(83.2)	37,912(79.2)	**11.96 (0.004)**	1197(86.8)	70,695(78.8)	**48.65 (< 0.001)**
	Female	151(14.4)	5638(21.2)		220(16.8)	10,282(20.8)		191(13.2)	19,671(21.2)	
Access to Media	Yes	516(55.9)	16,075(62.0)	14.278 (0.003)	669(56.0)	29,839(62.4)	**20.194 (< 0.001)**	701(55.0)	56,977(63.7)	**41.98 (< 0.001)**
	No	503(44.1)	9899(38.0)		606(44.0)	18,249(37.6)		682(45.0)	33,166(36.3)	
Husband/Partners Educational Status	No Formal Education	467(54.7)	7764(36.2)	117.27 (< 0.001)	585(56.5)	14,855(37.5)	**151.07 (< 0.001)**	650(55.0)	24,599(34.1)	**219.58 (< 0.001)**
	Primary Education	212(21.3)	6499(31.5)		238(20.7)	11,504(29.8)		284(21.8)	21,875(30.7)	
	Secondary Plus	224(24.1)	6949(32.3)		268(22.8)	13,053(32.7)		306(23.2)	25,926(35.2)	
Birth Order	First	195(19.1)	5646(21.9)	10.06 (0.023)	266(20.1)	10,567(22.0)	4.308 (0.183)	231(16.9)	19,282(21.5)	**21.780 (< 0.001**)
	2–4	471(44.4)	12,120(46.3)		603(46.4)	22,696(47.0)		662(46.9)	42,622(47.0)	
	5 +	355(36.4)	8273(31.4)		413(33.5)	14,931(31.0)		495(36.1)	28,462(31.4)	
Twin Status	Single Birth	995(97.2)	25,249(97.0)	0.11 (0.811)	1240(96.5)	46,770(97.0)	1.26 (0.419)	1346(97.1)	87,768(97.1)	0.00 (0.998)
	Multiple Births	26(2.8)	790(3.0)		42(3.5)	1424(3.0)		42(2.9)	2598(2.9)	
Parity	One birth	160(15.9)	4754(18.5)	**14.429 (0.015**)	211(16.0)	8754(18.3)	9.16 (0.088)	158(11.6)	14,614(16.3)	**29.945 (< 0.001)**
	Two births	163(16.2)	4822(18.5)		235(19.4)	9093(18.9)		258(19.3)	17,046(19.1)	
	Three births	150(14.8)	4239(16.1)		196(14.6)	7902(16.3)		210(14.6)	14,901(16.4)	
	Four or more births	548(53.1)	12,224(46.9)		640(50.1)	22,445(46.5)		762(54.4)	43,805(48.2)	
Mother Covered by Health Insurance	Yes	26(3.1)	1832(7.4)	23.29 (< 0.001)	34(3.0)	3398(7.3)	**31.64 (< 0.001)**	36(2.2)	7374(8.5)	**62.90 (< 0.001)**
	No	919(96.9)	21,476(92.6)		1137(97.0)	40,229(92.7)		1274(97.8)	73,983(91.5)	
Getting Permission to go Hospital	Big problem	254(25.8)	4856(20.6)	14.41 (0.003)	316(25.1)	8811(19.7)	**20.19 (< 0.001)**	389(28.4)	15,734(19.0)	**68.19 (< 0.001)**
	Not a big problem	689(74.2)	18,506(79.4)		866(74.9)	35,029(80.3)		910(71.6)	64,570(81.0)	
Getting Money Needed for Treatment	Big problem	631(65.0)	14,171(60.4)	7.52 (0.031)	773(63.4)	25,723(58.4)	**11.67 (0.005)**	939(70.4)	47,746(58.8)	**66.40 (< 0.001)**
	Not a big problem	312(35.0)	9195(39.6)		409(36.6)	18,123(41.6)		360(29.6)	32,565(41.2)	
Distance to Health Facility	Big problem	490(49.0)	10,520(44.1)	8.35 (0.021)	610(48.7)	19,152(42.7)	**16.59 (0.001)**	700(50.5)	34,553(41.7)	**37.85 (< 0.001)**
	Not a big problem	452(51.0)	12,844(55.9)		571(51.3)	24,692(57.3)		597(49.5)	45,748(58.3)	
Child’s Age	0–11 months	131(23.1)	4240(26.9)	4.27 (0.338)	190(27.1)	7606(26.1)	2.021 (0.663)	163(21.3)	12,462(22.5)	1.41 (0.765)
	12–23 months	181(33.0)	5216(32.6)		220(30.0)	9497(32.4)		182(24.5)	13,702(24.4)	
	24–35 months	127(21.4)	3203(20.1)		161(20.9)	6022(20.5)		164(19.9)	11,516(20.6)	
	36–59 months	128(22.5)	3217(20.4)		168(22.0)	6147(21.0)		266(34.3)	18,098(32.5)	
Sex of the Child	Male	548(53.6)	13,529(52.1)	0.85 (0 .400)	687(52.6)	25,182(52.4)	0.02 (0.901)	711(51.5)	45,628(50.5)	.46 (0.533)
	female	473(46.4)	12,510(47.9)		595(47.4)	23,012(47.6)		677(48.5)	44,738(49.5)	
Size of the Child	Above average	404(42.3)	8965(36.7)	11.88 (0.013)	450(38.1)	16,422(36.2)	2.03 (0.475)	546(41.7)	30,329(36.7)	**13.72 (0.007)**
	Average	389(39.2)	10,364(42.3)		518(42.0)	20,079(44.0)		538(40.6)	36,818(44.6)	
	Below average	188(18.5)	5106(20.9)		248(19.9)	9063(19.8)		249(17.7)	15,596(18.8)	
Decision Making Ability	Yes	585(65.9)	15,474(71.1)	10.35 (0.010)	707(63.8)	28,756(71.0)	**26.24 (< 0.001)**	797(63.8)	54,941(73.7)	**55.67 (< 0.001)**
	No	310(34.1)	6209(28.9)		395(36.2)	11,646(29.0)		432(36.2)	19,481(26.3)	
Position on Domestic Violence	Not Against wife beating	169(16.0)	3805(16.0)	0.02 (0.970)	205(17.3)	6541(14.8)	6.05 (0.083)	202(13.9)	11,111(13.5)	0.141 (0.766**)**
	Against wife beating	829(84.0)	21,254(84.0)		1047(82.7)	40,117(85.2)		1165(86.9)	74,724(86.5)	

*TMP* Traditional Medicine Practitioner

^a^weighted percentage

### Data analysis

Data analysis was conducted using STATA version 14. We extracted the required dataset from each country’s data file. We cleaned and recoded the extracted datasets to maintain consistency in the variables across all countries. We merged all the datasets to generate pooled data [24, 33]. To account for the weighting and complex sampling design used in conducting the DHS, we employed the survey prefix command on STATA (svy) to conduct descriptive and inferential statistics. We represented categorical variables using unweighted frequencies and weighted percentages. Random effect meta-analysis using DerSimonian, and Laird estimator based on inverse variance weights [34] was employed to pool the overall prevalence of TMP utilisation for childhood illness in SSA. Additionally, regional specific estimates were generated through subgroup random effect meta-analysis. Forest plots were used to present the overall and region-specific estimates of TMP utilisation for childhood illness.

We conducted a bivariate analysis to determine individual and community-level factors significantly associated with TMP utilisation for childhood illness. We then conducted a two-level multivariate regression analysis to identify factors independently associated with TMP utilisation for childhood illness. The two-level modelling implies that women were nested within clusters (primary sampling units). Clusters were considered random effects to account for the unexplained variability at the community level. We fitted four models, and they include the empty model (model 0), model 1 (community-level variables), model 2(individual-level variables) and model 3 (combination of individual and community-level variables). Model 0 provides the variance of the outcome variable due to the clustering of the primary sampling units (PSUs) without the independent variables. We compared our models using the log-likelihood ratio (LLR), Akaike’s information criterion (AIC) and the Bayesian information criterion (BIC) tests. The model with the highest log-likelihood and lowest AIC and BIC was considered the best fit for our multivariate regression analysis (see Tables 3, 4, and 5). The multivariate regression analysis results were presented as an adjusted odd ratio (AOR) and their corresponding 95% confidence intervals (CI). Statistical significance was considered if *p* < 0.05. We assessed multicollinearity among independent variables using variance inflation factor (VIF), and we found no evidence of multicollinearity among the independent variables (see Additional File 1).

**Table 3:** Individual and community factors associated with TMP use for childhood illnesses among childbearing women in 32 African countries

		**Model 0**	**Model 1(community)**	**Model 11(individual)**	**Model 111 AOR (95%CI)**
**Characteristics**	**Variable**				
Place of Residence	Rural		**1.41(1.15–1.73)**		1.25(1.00- 1.56)
	Urban		1		1
Household Wealth	Poor		**1.42(1.16–1.72)**		1.22(0.98- 1.52)
	Middle		1.23(0.98–1.54)		1.18(0.93–1.51)
	Rich		1		**1**
Sex of Household Head	Male		**1.74(1.37–2.20)**		**1.64(1.27–2.11)**
	Female		1		**1**
Decision Making Ability	Yes		1		1
	No		**1.27(1.09–1.45)**		1.06(0.90–1.25)
Maternal Education	No formal education			**1.77(1.36–2.30)**	**1.62(1.23–2.12)**
	Primary education			1.11(0.87–1.43)	1.05(0.81–1.36)
	Secondary plus			**1**	1
Currently Working	Yes			**1.28(1.10–1.50)**	**1.25(1.06–1.47)**
	No			**1**	**1**
Access to Media	Yes			1	1
	No			**1.31(1.13–1.52)**	**1.19(1.02–1.39)**
Husband/Partners Educational Status	No Formal Education			**1.27(1.03–1.57)**	1.16(0.93–1.44)
	Primary Education			0.88(0.71–1.09)	0.82(0.66–1.01)
	Secondary Plus			1	1
Birth Order	First			1	1
	2–4			0.92(0.60–1.41)	0.87(0.57–1.34)
	5 +			0.71(0.45–1.14)	0.65(0.40–1.04)
Parity	One birth			1	1
	Two births			1.02(0.66–1.41)	1.05(0.68–1.63)
	Three births			1.01(0.61–1.65)	1.08(0.66–1.79)
	Four or more births			1.27(0.77–2.09)	1.39(0.84–2.29)
Covered by Health Insurance	Yes			1	1
	No			**2.21(1.47–3.33)**	**2.37(1.53–3.66)**
Getting Permission to go to the hospital	Big problem			**1.20(1.01–1.43)**	**1.23(1.03–1.47)**
	Not a big problem			1	1
Getting money Needed for Treatment	Big problem			1.05(0.89–1.24)	1.05(0.89–1.25)
	Not a big problem			1	1
Distance to Health Facility	Big problem			1.13(0.96–1.32)	1.08(0.92–1.28)
	Not a big problem			1	1
Size of the Child at Birth	Above average			**1.23(1.05–1.44)**	**1.20(1.03–1.41)**
	Below average			0.89(0.72–1.09)	0.91(0.74–1.12)
	Average			1	1
**Random effect result**					
	PSU variance (95% CI)	0.35(0.25–0.48)	0.38(0.27–0.54)	0.41(0.29–0.57)	0.41(0.29–0.58)
	ICC	0.09	0.10	0.11	0.11
	LR Test	79.99, p < 0.001	76.14, p < 0.001	73.67, p < 0.001	66.61, p < 0.001
	Wald chi-square	Reference	79.24	188.03	198.46
**Model fitness**					
	Log Likelihood	-4307.591	-3684.198	-3349.869	-3219.234
	AIC	8619.182	7382.397	6737.737	6486.468
	BIC	8635.593	7438.57	6888.077	6675.706

*PSU* Primary Sampling Units, *ICC* Interclass correlation Coefficient, *TMP* Traditional medicine Practitioner, *LR Test* Likelihood ratio Test, *AIC* Akaike’s Information Criterion, *BIC* Bayesian information criterion

Model 0 is the null model, a baseline model without any independent variable

Model 1 is adjusted for community level variables

Model 2 is adjusted for individual level variables

Model 3 is the final model adjusted for community and individual level variables

**Table 4:** Individual and community factors associated with TMP use for Diarrhea among childbearing women in 32 African countries

		**Model 0**	**Model 1**	**Model 11**	**Model 111 AOR (95%CI)**
**Characteristics**	**Variable**				
Place of Residence	Rural		**1.26(1.06–1.50)**		1.15(0.95–1.40)
	Urban		1		1
Household Wealth	Poor/		**1.54(1.29–1.83)**		**1.33(1.10–1.61)**
	Middle		1.19(0.98–1.46)		1.12(0.90–1.39)
	Rich		**1**		1
Sex of Household Head	Male		**1.32(1.09–1.60)**		**1.25(1.02–1.54)**
	Female		**1**		1
Decision Making Ability	Yes		1		1
	No		**1.32(1.16–1.50)**		1.11(0.96–1.27)
Maternal Education	No formal education			**1.40(1.12–1.76)**	**1.30(1.03–1.65)**
	Primary education			1.11(0.90–1.38)	1.06(0.84–1.32)
	Secondary plus			1	1
Access to Media	Yes			1	1
	No			**1.29(1.13–1.47)**	**1.17(1.02–1.34)**
Husband/Partners Educational status	No Formal Education			1.41(1.16–1.70)	**1.26(1.04–1.53)**
	Primary Education			0.91(0.75–1.11)	0.84(0.69–1.02)
	Secondary Plus			1	**1**
Covered by Health Insurance	Yes			1	1
	No			**2.46(1.66–3.62)**	**2.41(1.63–3.59)**
Getting Permission to go to the Hospital	Big problem			1.26(1.08–1.47)	**1.29 (1.10–1.51)**
	Not a big problem			1	1
Getting Money needed for Treatment	Big problem			1.00(0.86–1.16)	1.01(0.87–1.18)
	Not a big problem			1	1
Distance to Health Facility	Big problem			**1.17(1.01–1.34)**	1.12(0.96–1.29)
	Not a big problem			1	1
**Random effect result**					
	PSU variance (95% CI	0.27(0.19–0.38)	0.30(0.21–0.42)	0.29(0.20–0.42)	0.29(0.20–0.42)
	ICC	0.08	0.08	0.08	0.08
	LR Test	71.29, *p* < 0.001	68.78, *p* < 0.001	55.66, *p* < 0.001	53.09, *p* < 0.001
	Wald chi-square	Reference	91.27	172.69	187.82
**Model fitness**					
	Log-likelihood	-5912.830	-5004.559	-4520.899	-4391.942
	AIC	11,829.66	10,023.12	9063.799	8815.883
	BIC	11,847.28	10,083.55	9157.682	8952.06

*PSU* Primary Sampling Units, *ICC* Interclass correlation Coefficient, *TMP* Traditional medicine Practitioner, *LR Test* Likelihood ratio Test, *AIC* Akaike’s Information Criterion, *BIC* Bayesian information criterion

Model 0 is the null model, a baseline model without any independent variable

Model 1 is adjusted for community level variables

Model 2 is adjusted for individual level variables

Model 3 is the final model adjusted for community and individual level variables

**Table 5:** Individual and community factors associated with TMP use for Fever /Cough among childbearing women in 32 African countries

		**Model 0 AOR (95%CI)**	**Model 1 AOR (95%CI)**	**Model 11 AOR (95%CI)**	**Model 111 AOR (95%CI)**
**Characteristics**	**Variable**				
Place of Residence	Rural		**1.38(1.16–1.63)**		1.18(0.98–1.43)
	Urban		1		1
Household Wealth	Poor		**1.60(1.36–1.89)**		**1.30(1.08–1.57)**
	Middle		**1.43(1.18–1.72)**		**1.29(1.05–1.58)**
	Rich		1		**1**
Sex of Household Head	Male		**1.67(1.37–2.03)**		**1.56(1.26–1.93)**
	Female		1		1
Decision Making Ability	Yes		1		1
	No		**1.44(1.28–1.63)**		**1.17(1.03–1.34)**
Maternal Education	No formal education			**1.75(1.41–2.17)**	**1.55(1.23–1.94)**
	Primary education			1.05(0.86–1.30)	0.95(0.76–1.17)
	Secondary plus			1	1
Currently Working	Yes			**1.31(1.15–1.50)**	**1.28(1.11–1.47)**
	No			1	1
Access to media	Yes			1	1
	No			**1.24(1.10–1.41)**	1.12(0.99–1.28)
Husband/Partners Educational Status	No Formal Education			**1.37(1.15–1.64)**	**1.29(1.07–1.55)**
	Primary Education			0.91(0.78–1.08)	0.87(0.72–1.05)
	Secondary Plus			1	1
Birth Order	First order			1	1
	2–4 order			1.06(0.79–1.43)	1.07(0.78–1.45)
	5plus order			0.94(0.67–1.32)	0.91(0.64–1.28)
Parity	one			1	1
	two			1.22(0.88–1.69)	1.24(0.89–1.74)
	three			1.02(0.69–1.49)	1.08(0.73–1.60)
	Four or more			1.16(0.79–1.70)	1.22(0.83–1.80)
Covered by Health Insurance	Yes			1	1
	No			**2.77(1.93–3.99)**	**2.90(1.96–4.27)**
Getting Permission to go to the Hospital	Big problem			**1.34(1.17–1.54)**	**1.36(1.18–1.57)**
	Not a big problem			1	1
Getting Money Needed for Treatment	Big problem			**1.31(1.13–1.52)**	**1.29(1.11–1.50)**
	Not a big problem			1	1
Distance to Health Facility	Big problem			1.14(1.00–1.30)	1.10(0.96–1.26)
	Not a big problem			1	1
Size of Child	Above average			**1.22(1.07–1.39)**	**1.19(1.04–1.36)**
	Below Average			1.02(0.86–1.21)	1.03(0.87–1.22)
	Average			1	1
**Random effect result**					
	PSU variance (95% CI)	0.40(0.30–0.52)	0.37(0.27–0.49)	0.44(0.33–0.58)	0.43(0.32–0.58)
	ICC	0.11	0.10	0.12	0.12
	LR Test	146.16, *p* < 0.001	112.79, *p* < 0.001	128.13, *p* < 0.001	116.49, *p* < 0.001
	Wald chi-square	Reference	155.75	365.85	385.70
**Model fitness**					
	Log-likelihood	-7121.819	-6139.199	-5602.321	-5356.455
	AIC	14,247.64	12,292.4	11,242.64	10,760.91
	BIC	14,266.49	12,357.04	11,416.21	10,979.48

*PSU* Primary Sampling Units, *ICC* Interclass correlation Coefficient, *TMP* Traditional medicine Practitioner, *LR Test* Likelihood ratio Test, *AIC* Akaike’s Information Criterion, *BIC* Bayesian information criterion

Model 0 is the null model, a baseline model without any independent variable

Model 1 is adjusted for community level variables

Model 2 is adjusted for individual level variables

Model 3 is the final model adjusted for community and individual level variables

### Ethical clearance

Ethical clearance was not obtained since we used secondary data that is publicly available. However, ethical clearance was obtained prior to conducting the various surveys from the Ethics Committee of ORC Macro Inc. and the Ethics Boards of partner organisations of various countries, such as the Ministries of Health. Further details regarding DHS data usage and ethical standards can be accessed via the following link https://dhsprogram.com/methodology/Protecting-the-Privacy-of-DHS-Survey-Respondents.cfm.

## Results

### Distribution of the individual and community factors and the use of TMPs for childhood illness in SSA

Among women who sought the service of a TMP for childhood illness, close to half were between the ages of 25–34 years (*n* = 461,44.2%), and more than half had no formal education (*n* = 576, 59.0%). Also, majority of women who used the service of a TMP for childhood illness lived in a rural setting (*n* = 836,82.2%),and households headed by a man(*n* = 870,85.6%) and did not have health insurance (*n* = 919,96.9%). Similar patterns were observed regarding consultation of a TMP for diarrhea and cough/fever (Table 2).

### Prevalence of TMP services for childhood illness in sub-Saharan Africa

**Fig. 1:**
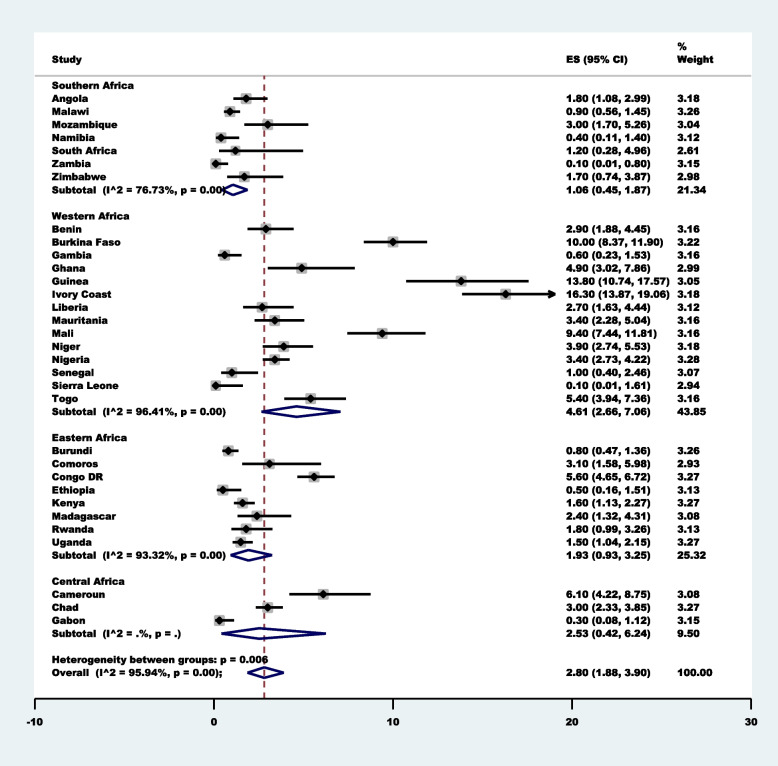
Forest plot showing the prevalence of the use of traditional medicine practitioners for childhood illness in 32  sub-Saharan African Countries

Approximately 3 in 100 [2.80% (95%CI: 1.88–3.90)] women who sought healthcare for childhood illnesses utilised the service of a traditional medicine practitioner, with the highest seen in Ivory Coast (16.30% (95%CI: 13.87–19.06)] followed by Guinea (13.80% (95%CI: 10.74–17.57)] with the lowest seen in Sierra Leone [0.10%(95%CI:0.01–1.61)] (Fig. 1). Sub-regional differences indicated that West Africa had the highest prevalence [4.61%; 95%CI: 2.66–7.06)] and Southern Africa had the lowest [1.06%;95%CI:0.45–1.87)].

**Fig. 2:**
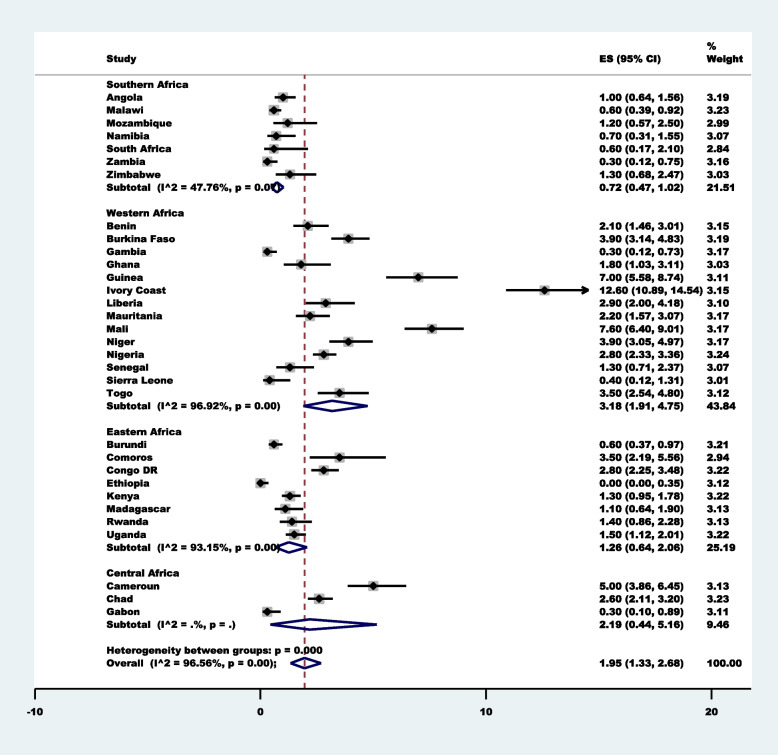
Forest plot showing the prevalence of the use of traditional medicine practitioners for diarrhea in 32  sub-Saharan African Countries

**Fig. 3:**
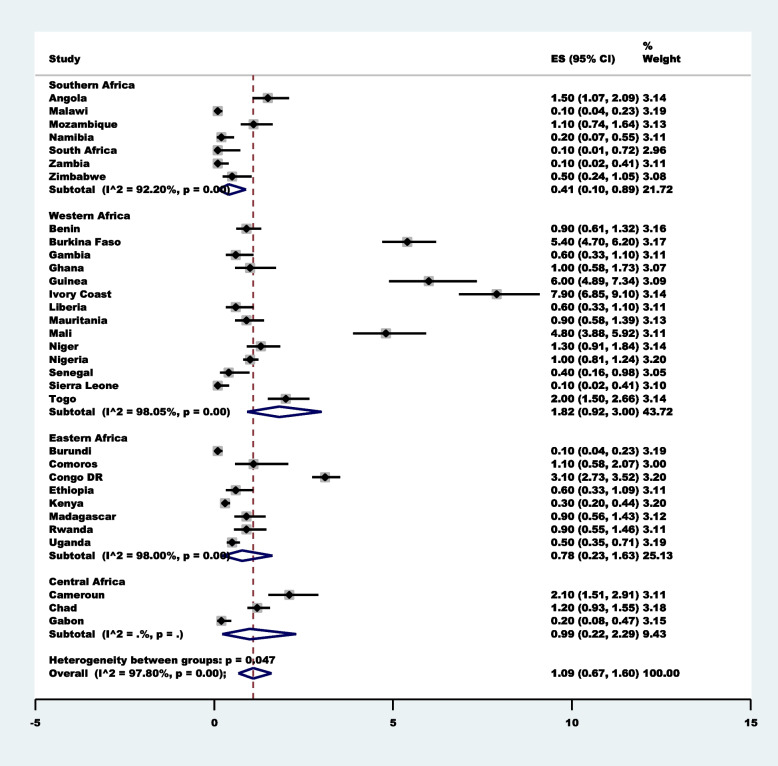
Forest plot showing the prevalence of the use of traditional medicine practitioners for fever/cough in 32 sub-Saharan African Countries

Approximately 2 in 100 [1.95% (95%CI: 1.33–2.68)] women sought healthcare for childhood diarrhea. Ivory Coast [12.60% (95%CI: 10.89–14.54)] had the highest prevalence, followed by Mali [7.60% (95%CI: 6.40–9.01)] and Guinea [7.00% (95%CI: 5.58–8.74)] (Fig. 2). Western Africa [3.18% (95%CI: 1.91–4.75)] and Southern Africa [0.72%;95%CI:0.47–1.02)] had the highest and lowest prevalence, respectively. The prevalence of the use of TMP for fever/cough is summarised in Fig. 3, and it indicates that 1 in every 100 women who sought healthcare for childhood Cough/fever sought the service of a traditional medicine practitioner [1.09% (95%CI: 0.67–1.60)]. Similarly, Cote d’Ivoire [7.90%;95%CI:6.85–9.10)] had the highest prevalence, followed by Guinea [6.00%;95%CI:4.89–7.34)]. Also, Western Africa [1.82%;95%CI:0.92–3.00)] had the highest prevalence compared to other SSA regions.

### Factors associated with the use of traditional medicine practitioners for childhood illness in sub-Saharan Africa

Table 3 shows a mixed effect multilevel modelling results of the individual and community factors associated with the use of traditional medicine practitioners for childhood illness. Women with no formal education [AOR = 1.62;95%CI:1.23–2.12], those who lived in households headed by a male [AOR = 1.64;95%CI:1.27–2.11], without access to media [AOR = 1.19; 95%CI:1.02–1.39], those without health insurance [AOR = 2.37;95%CI: 1.53–3.66] those who considered it a problem getting permission to visit a health facility[AOR = 1.23;95%CI:1.03–1.47] and perceived the size of their children at birth to be above average[AOR = 1.20;95%CI:1.03–1.41] were more likely to seek TMPs’ services than their respective counterparts.

In terms of the use of TMP for childhood diarrhea (Table 4), our analysis indicated that women with no formal education [AOR = 1.30;95%CI:1.03–1.65), those whose husbands had no formal education [AOR = 1.26;95%CI:1.04–1.53], those from poor households [AOR = 1.33;95%CI:1.10–1.61] and without health insurance [AOR = 2.41;95%CI:1.63–3.59) were more likely than their respective counterparts to use TMP services.

Table 5 summarises the correlates of TMP’s services used for cough/fever among children. We observed that women without formal education [AOR = 1.55;95%CI:1.23–1.94], those in the poor [AOR = 1.30;95%CI:1.08–1.57], and middle [AOR = 1.29;95%CI:1.05–1.58] wealth quantiles, those residing in male-headed households [AOR = 1.56;95%CI:1.26–1.93], and those whose husband/partner had no formal education [AOR = 1.29;955CI:1.07–1.55] had higher odds of using a TMP to treat their children’s fever/cough. Also, women not covered by health insurance [AOR = 2.90;95%CI: 1.96–4.27] had challenges getting permission to seek medical care[1.36;95%CI: 1.18–1.57], getting money needed for treatment [AOR = 1.29;95%CI: 1.11–1.50]whose perceived child size at birth was above average at birth [AOR = 1.19;95%CI:1.04–1.36] and who has no input in household decision making [AOR = 1.17;95%CI:1.03–1.34] were of higher odds to seek the service of a traditional medicine practitioner to treat their childhood cough/fever.

## Discussion

Child survival remains a critical public health issue in SSA despite the improvements made over the years [1]. TM is widely used to manage communicable and non-communicable diseases in SSA [8]. The role of traditional and complementary medicine, especially TM practitioners, in managing childhood illness has not been fully explored in this setting. Our study provides the first-ever insight into TMPs’ contributions to managing childhood illness at a regional level. Specifically, our study examined the proportion of women with children less than five years old who sought the service of a traditional medicine practitioner to treat their children’s illness (diarrhea or fever/cough or both) as well as individual and community factors associated with such a pattern of use in using DHS data from 32 African countries.

Our study indicates that 3% of women who sought healthcare for childhood illnesses used the services of TMPs. Our finding is consistent with a national representative study conducted in China, Mexico, Russia, South Africa, and Ghana [35], which found that the rate of use of TMP was 1.7% for South Africa and 1.5% for Ghana [35]. A similar prevalence has been reported in a similar study conducted in Indonesia in which TMP utilisation rate in childhood illness treatment was 3.4% in the month preceding the survey [36]. Another South African study reported similar findings in which 2.5% of survey respondents reported visiting a TMP when sick, and 3.3% of respondents reported seeking any form of health services consulted a traditional healer [11]. An Ethiopian community-based study reported that 3.3% of mothers visited a Traditional Birth Attendant (TBA) when their child was sick [16].

On the other hand, our finding was lower than the 26.4% average reported in 32 countries majority of which were in Europe and Asia [37]. The only African country included in this multicounty study was South Africa, and the reported prevalence of TM provider use was 24% which is still higher than the pooled prevalence reported in our study [37]. A previous systematic review on TCM use in sub-Saharan Africa reported that traditional medicine practitioner utilisation rate ranges from 1.2%–67%, with a lower rate (1.2%–44.1%) observed among studies that utilised a larger sample size than those that utilised smaller samples (37.5%–67%) [8].

The pooled prevalence of TMP use for childhood diarrhea was 2% in our study. Our finding is lower than what was reported in a systematic review in developing countries in which a seven median percent of mothers/caregivers used the service of traditional medicine practitioners [38]. Even though Ivory Coast and Mali show relatively high prevalence regarding the use of traditional medicine practitioners to treat childhood diarrhea, community studies in these countries and other African countries reported higher use of traditional medicine practitioners. For instance, a community cross-sectional study in Mali found that 57% of parents/guardians sought the service of TMP [15], and 6.4% of caregivers visited a traditional medicine practitioner in the rural areas of Burkina Faso [17]. In comparison, 11.3% of children with diarrhea sought the services of a traditional medicine practitioner [12].

Regarding fever/cough, one in every 100 women who sought care for her child’s fever/cough visited a traditional medicine practitioner, with Ivory Coast and Mali reporting the highest proportions of women who visited a traditional medicine practitioner. Compared with community-based surveys, our overall prevalence is lower, including the TMP utilisation rates of individual countries considered in our study. For example, a community study in Benin and Gabon reported that 7% and 18% of mothers chose a traditional healer as their first healthcare provider, respectively [19]. A similar study in Ethiopia found that 18.5% of parents/guardians first consulted with a TMP for their children's fever [18].

The overall low TMP utilisations rates for diarrhea and fever/cough observed in our study may reflect that TMPs are not the preferred healthcare providers for these conditions; instead, conventional medical care is the mainstay as it has been reported in several multinational studies assessing the healthcare seeking behaviour for childhood illness in Africa [7, 22, 23, 27]. It could also be due to lack of disclosure to the interviewer regarding their consultation with a TMP, given that non -disclosure of TM use by the general public is widespread in Africa [8]. Over the years, there has been an increased child survival intervention from governments and international organisations, which promote the use of formal healthcare options to manage childhood diseases, and this may help explain our finding [39, 40]. Also, the current barriers to TM use in Africa may explain the low consultation of a TMP. These include the lack of belief in the safety and efficacy of TM due to the absence of scientific evidence to support TM practice, absence/limited regulation of TM practice, perceived lack of education and training of TMP in the African region and the association of TM with witchcraft and sorcery [8]. It is important to note that the low use of TMP for childhood illness in our study does not imply that TM plays an insignificant role in managing childhood illness in Africa. Current evidence suggests that most mothers/caregivers self-medicate with home remedies, TM products, or conventional products bought from the market or sourced from friends or relatives before seeking care outside the home [29, 31, 32, 41]. Thus, our study's low TMP utilisation rate may be explained by high self-medication practices with TM products or in combination with conventional products, not only among children but also among adults [8]. Also, we observed a higher prevalence rate in Ivory Coast and Mali and the West African region than in other countries and regions. Such relatively high use may reflect the extent of TM and recognition and its integration into the healthcare system in these countries compared to other countries and regions. For instance, as of December 2018, most countries in West Africa, including Ivory Coast and Mali, have met most of the indicators for integrating TM into national health systems [10].

Results from our multilevel modelling indicate that women with no formal education were more likely to seek the service of a TMP for their sick children. Similarly, those without media access had higher odds of seeking the service of a TMP. Education has a role in enlightening women about the efficacy and safety of formal healthcare use as opposed to consulting with a TMP, which might explain our finding. Similar findings have been reported in a household survey on TMP consultation for childhood diseases in south-eastern Nigeria [12], the Amhara region of Ethiopia [28], a systematic review on traditional and complementary medicine use among the general population in sub-Saharan Africa [8], a survey conducted among the adult population in China, India, Ghana, Mexico, Russia and South Africa [35] and a mixed method study among African migrant women in Sydney, Australia [42]. However, our finding contrasted with studies reporting complementary and alternative medicine use among children in Germany [43] and Italy [44] and among the adult population in Western countries, including Brazil, in which higher education status was associated with seeking the service of a TMP [45, 46]. The disparity in the targeted population, literacy rates in these regions, and what constitutes traditional and complementary medicine may explain the difference between educational status and TMP consultation reported in our study and those reported in Western countries.

In line with community-based African studies on the use of TMP for childhood illness [28], our study revealed that women from poor households had higher odds than their wealthy counterparts of seeking the service of TMPs for their children’s illness. Similar findings were reported among adults in Ghana and India and in a systematic review of traditional and complementary medicine users’ characteristics in sub-Saharan Africa [8, 35]. However, a systematic review on traditional and complementary medicine use among children outside of Africa gives an opposite picture concerning our finding [30]. Since TM is considered affordable due to its relatively low cost compared to conventional medicine in Africa might explain why women from low socio-economic households were likely to seek care from TMP for their children [8]. A South African study on the cost of TM use for non-communicable diseases reported that most participants spent little to nothing to access TM [47].

We also observed that women without health insurance were more likely to seek care from TMP for their children. Our finding is consistent with a nationwide study among older Ghanaians in which TM was primarily used by those who were not insured [48]. However, another Ghanaian study in two districts of the Ashanti region found no significant association between health insurance status and TM [49]. Health insurance enables access to healthcare services since it provides financial risk protection and decreases healthcare expenditure. However, most health insurance schemes do not cover TM, and the majority of the population in Africa is uninsured [50, 51]. Therefore, uninsured women in our study do not have the financial protection associated with seeking modern healthcare and will seek TM, which is considered less expensive.

Women who reside in households headed by a male had higher odds than a female of seeking treatment from a TMP for their childhood illness, especially fever/cough. Our finding contrasted with the finding from a multi-country study using DHS data that focussed on the utilisation of conventional healthcare for childhood illness in which living in a household headed by a man was associated with conventional healthcare [7]. However, individual country studies found that the odds of utilising conventional healthcare for childhood illness were higher when the household head was a woman [52, 53]. Our regression analysis indicates that women who were not involved in decision-making at home and had issues getting permission to visit the hospital were more likely to seek care for their child’s fever/cough from a TMP. Such a finding seems to suggest that the decision to utilise TMP services for fever/cough is often in the hands of the husband/partner or someone else, which further supports our previous finding on the likely consultation with a TMP if the head of the household is male. The patriarchal nature of most African societies may help explain our findings. Patriarchy in Africa prevents women from having power and influence in society compared to men [54]. As such, women are often unable to make decisions on education, finances, and the health of their families, as their male counterparts often play such a role. The use of the service of a TMP for childhood fever/cough was more likely if the perceived size of the child was above average than if it was average. It is possible that the parent/caregiver considered the above-average size of their child to be linked to supernatural causes rather than biomedical, and as such only TMPs competent to manage such condition [55, 56].

### Policy and Practice Implication

Our findings suggest that the TMPs continue to have an important role in managing childhood illness in SSA. Thus, policymakers and health service providers need to consider the potential role of TMPs in child health policies and interventions through TMP training and promoting collaboration between TMP and conventional healthcare providers to achieve better child health outcomes.

The identified factors associated with TMP consultation underscore that women and children with such characteristics are considered risk factors for TMP consultation for childhood illness in SSA. Thus, women and communities with such characteristics, including low socio-economic backgrounds, must be prioritised when designing and implementing child health interventions in SSA. It is also important that child health policies and interventions are incorporated into women’s empowerment as a tool to enhance women’s active contribution to decision-making regarding the health of their children.

### Strengths and limitation

A key strength of our study is that it uses nationally representative data from 32 SSA countries. Our findings can, therefore, be generalised to all children in these countries. Also, selection bias was reduced since a multi-stage sampling strategy was employed in conducting the DHS in all 32 countries. DHS uses a standardised questionnaire, and data collection is done by trained personnel, which adds rigour to our findings. We employed vigorous analysis using multilevel modelling to account for the varied clusters in our data.

Notwithstanding these strengths, readers should consider some limitations when interpreting our findings. First, casual relationships cannot be inferred due to the cross-sectional design employed in our study. Second, our analysis relied on the variables in the DHS dataset to determine the factors associated with TMP use for childhood disease. Thus, we could not, include other factors that may influence health-seeking behaviour for childhood illnesses, such as women’s knowledge of childhood disease and health workers’ attitudes, given that these variables were not captured in the DHS datasets used in our study. We cannot rule out the potential for social desirability and recall biases since some of our study data were collected based on retrospective self-report.

## Conclusions

Our findings indicate that approximately three in every 100 women surveyed in the 32 African countries patronised TMP services for childhood diarrhea and fever/cough. Factors such as the educational status of parents, media access, household wealth, maternal health insurance status, sex of household head, and maternal decision-making ability to access healthcare were significantly associated with TMP utilisation for diarrhea and fever/cough. Our findings suggest that TMPs continue to have an important role in managing childhood illness in SSA. It is imperative that policymakers and service providers take into account the potential role of TMPs in policies and interventions for child health. Also, efforts to improve child health in SSA should consider the characteristics of women who use TMPs for childhood diseases identified in our study.

## Supplementary Information

**Additional file 1.**

## Data Availability

The datasets generated and analysed during the current study are freely available on the DHS website https://dhsprogram.com/data/available-datasets.cfm.

## Abbreviations

AIC: Akaike’s Information Criterion
AOR: Adjusted Odd Ratio
BIC: Bayesian Information Criterion
DHS: Demographic and Health Surveys
ICC: Interclass Correlation Coefficient
LLR: Log-likelihood Ratio
PSU: Primary Sampling Units
TMP: Traditional Medicine Practitioner
TM: Traditional Medicine
SSA: Sub-Saharan Africa
SDG: Sustainable Development Goals
