# Supplementary material for: The use of traditional medicine practitioner services for childhood illnesses among childbearing women: a multilevel analysis of demographic and health surveys in 32 sub-Saharan African countries
Source: BMC Complement Med Ther. 2023 Apr 29;23:137. doi: 10.1186/s12906-023-03972-3 (PMC10148432; doi:10.1186/s12906-023-03972-3)
Supplement: Supplementary file 1 — Additional file 1. [file 12906_2023_3972_MOESM1_ESM.docx]

**Collinearity Statistics for TMP use for childhood** **illness**

| **Variables** | **Tolerance** | **VIF** |
| --- | --- | --- |
| Place of Residence | 0.715291 | 1.40 |
| Wealth Quantile | 0.691547 | 1.45 |
| Sex of Household Head | 0.981646 | 1.02 |
| Decision-Making Ability | 0.935023 | 1.07 |
| Mother Education Status | 0.584596 | 1.71 |
| Respondent Currently Working | 0.968578 | 1.03 |
| Access to Media | 0.860538 | 1.16 |
| Husband Education | 0.640496 | 1.56 |
| Covered by Health Insurance | 0.966211 | 1.03 |
| Getting Medical Help for Self: Getting Permission to go to a healthcare facility | 0.852373 | 1.17 |
| Getting Medical Help for Self: Getting Money Needed For Treatment | 0.792387 | 1.26 |
| Getting Medical Help for Self: Distance to Health Facility | 0.792029 | 1.26 |
| Birth order | 0.302989 | 3.30 |
| Parity | 0.300914 | 3.32 |
| Size of the child at birth | 0.995061 | 1.00 |
| Mean VIF |  | 1.52 |

**Collinearity Statistics for TMP use for Diarrhoea**

| **Variables** | **Tolerance** | **VIF** |
| --- | --- | --- |
| Place of Residence | 0.713502 | 1.40 |
| Wealth Quantile | 0.683794 | 1.46 |
| Sex of Household Head | 0.985582 | 1.01 |
| Decision-Making Ability | 0.938254 | 1.07 |
| Mother Education Status | 0.601230 | 1.66 |
| Access to Media | 0.858928 | 1.16 |
| Husband Education | 0.633543 | 1.58 |
| Covered by Health Insurance | 0.965301 | 1.04 |
| Getting Medical Help for Self: Getting Permission to go to a healthcare facility | 0.846442 | 1.18 |
| Getting Medical Help for Self: Getting Money Needed For Treatment | 0.776910 | 1.29 |
| Getting Medical Help for Self: Distance to Health Facility | 0.776905 | 1.29 |
| Mean VIF |  | 1.29 |

**Collinearity Statistic for TMP use for Cough/fever**

| **Variables** | **Tolerance** | **VIF** |
| --- | --- | --- |
| Place Of Residence | 0.709906 | 1.41 |
| Wealth Quantile | 0.676697 | 1.48 |
| Sex of Household Head | 0.983555 | 1.02 |
| Decision-Making Ability | 0.933857 | 1.07 |
| Mother Education | 0.564764 | 1.77 |
| Respondent Currently Working | 0.967136 | 1.03 |
| Access to Media | 0.846857 | 1.18 |
| Husband Education | 0.622389 | 1.61 |
| Covered by Health Insurance | 0.956411 | 1.05 |
| Getting Medical Help for Self: Getting Permission to go to a health facility | 0.847170 | 1.18 |
| Getting Medical Help for Self: Getting Money Needed for Treatment | 0.780607 | 1.28 |
| Getting Medical Help for Self: Distance to Health Facility | 0.781035 | 1.28 |
| Birth Order | 0.334078 | 2.99 |
| Parity | 0.333533 | 3.00 |
| Size of Child | 0.996945 | 1.00 |
| Mean VIF |  | 1.49 |
